# Complete Genome Sequence of ZG49, a T7-Like Bacteriophage Lytic to *Escherichia coli* Isolates

**DOI:** 10.1128/genomeA.01304-17

**Published:** 2018-01-25

**Authors:** Hongyan Shi, Zhonghe Guo, Yangyang Liu, Yuchong Hao, Jinghua Li, Yanbo Sun

**Affiliations:** a Department of Pathogen Biology, College of Basic Medical Sciences, Jilin University, Changchun, People’s Republic of China

## Abstract

Here, we describe the complete genome sequence of the *Escherichia coli* bacteriophage ZG49, isolated from a sewage sample. ZG49 is a linear double-stranded DNA T7-like podophage, with a genome of 40,291 bp, containing 44 predicted open reading frames.

## GENOME ANNOUNCEMENT

Bacteriophage ZG49 was isolated from a sewage sample and is capable of infecting *Escherichia coli*. *E. coli* is frequently found in the gastrointestinal tract of many animals and is considered to be a causative pathogen of foodborne diseases (1). An investigation of the phage features and genomics might help develop phage-based products, including phage-based food additives that protect food from *E. coli* (2, 3). In the present study, a virulent bacteriophage, ZG49, was isolated from sewage samples using a strain of clinically isolated *E. coli*. This phage was able to infect 13 of 42 *E. coli* clinical isolates and to form clear plaques on a lawn of host cells. It was categorized as a member of the family *Podoviridae*, according to morphological features, by transmission electron microscopy. In a one-step growth test, phage ZG49 was shown to have a latent period of 8 min, with a corresponding burst size of 150 PFU/cell. These characteristics of phage ZG49 let us completely sequence its genome.

Genomic DNA of ZG49 was extracted and purified as described previously (4). Whole-genome sequencing was performed with an Illumina MiSeq sequencing platform at Sangon Biotech Co., Ltd. (Shanghai, China). As a result, a total of 1,402,295 reads (1,403,742 raw reads) were trimmed and assembled using SPAdes (5) and GapFiller (BaseClear). The complete genome of phage ZG49 is a linear double-stranded DNA (dsDNA) of 40,291 bp, with 401-bp long terminal repeats. The G+C content of the genome was 49.8%. Running a BLASTN search with whole genomes revealed that it has similarity to other *Escherichia* phage genomes, including those of *Escherichia* phage LM33_P1 (GenBank accession number LT594300), with 83% query coverage and 96% identity; *Escherichia* phage PE3-1 (GenBank accession number KJ748011), with 85% query coverage and 95% identity; *Escherichia* phage JSS1 (GenBank accession number KX689784), with 85% query coverage and 95% identity; and *Enterobacter* phage K1F (GenBank accession number AM084414), with 84% query coverage and 95% identity, respectively. Genome annotations were conducted with the Rapid Annotation using Subsystem Technology (RAST) server (6). A total of 44 proteins were predicted in the phage ZG49 genome, including 27 proteins that are functionally annotated and 17 that are hypothetical proteins. Morphology and genome analyses suggest that the phage ZG49 is a T7-like podophage. The functionally annotated proteins related to T7-like phage include DNA-directed RNA polymerase (EC 2.7.7.6), T7-like phage single-stranded DNA (ssDNA)-binding protein, T7-like phage endonuclease (EC 3.1.21.2), T7-like phage primase/helicase protein, T7-like phage DNA polymerase (EC 2.7.7.7), T7-like phage head-to-tail joining protein, T7-like tail tubular protein A, and T7-like tail tubular protein B.

### Accession number(s).

The complete genome sequence of *E. coli* phage ZG49 is available in GenBank under the accession number KX669227. The version described here is KX669227.5.
